# A Novel Mutation in the *Complement Component 3* Gene in a Patient with Selective IgA Deficiency

**DOI:** 10.1007/s10875-012-9775-z

**Published:** 2012-09-21

**Authors:** Elisangela Santos-Valente, Ismail Reisli, Hasibe Artaç, Raphael Ott, Özden Sanal, Kaan Boztug

**Affiliations:** 1 CeMM Research Center for Molecular Medicine of the Austrian Academy of Sciences, Lazarettgasse 14, AKH BT 25.3, A-1090 Vienna, Austria; 2 Meram Medical Faculty, Department of Pediatric Immunology, Necmettin Erbakan University, Beysehir Yolu, 42080 Konya, Turkey; 3 Department of Pediatric Immunology and Allergy, Selçuk University Selçuklu Medical Faculty, Alaeddin Keykubat Kampusu, 42075 Konya, Turkey; 4 Immunology Division, Hacettepe University, Children’s Hospital, 06100 Sihhiye, Ankara, Turkey; 5 Department of Pediatrics and Adolescent Medicine, Medical University of Vienna, Währinger Gürtel 18-20, A-1090 Vienna, Austria

**Keywords:** Complement deficiency, complement component 3, *Streptococcus pneumoniae*, primary immunodeficiency

## Abstract

**Purpose:**

Immunological and molecular evaluation of a patient presenting with recurrent infections caused by *Streptococcus pneumoniae* and low complement component 3 (C3) levels.

**Methods:**

Immunological evaluation included complement components and immunoglobulin level quantification as well as number and function of T cells, B cells and neutrophils. Serotype-specific immunoglobulin G antibodies against *S. pneumoniae* capsular polysaccharides were quantified by ELISA in serum samples before and after vaccination with unconjugated polysaccharide vaccine. For the molecular analysis, genomic DNA from the patient and parents were isolated and all exons as well as exon-intron boundaries of the *C3* gene were sequenced by Sanger sequencing.

**Results:**

A 16-year-old male, born to consanguineous parents, presented with recurrent episodes of pneumonia caused by *S. pneumoniae* and bronchiectasis. The patient showed severely reduced C3 and immunoglobulin A levels, while the parents showed moderately reduced levels of C3. Mutational analysis revealed a novel, homozygous missense mutation in the *C3* gene (c. C4554G, p. Cys1518Trp), substituting a highly conserved amino acid in the C345C domain of C3 and interrupting one of its disulfide bonds. Both parents were found to be carriers of the affected allele. Vaccination against *S. pneumoniae* resulted in considerable clinical improvement.

**Conclusions:**

We report a novel homozygous mutation in the *C3* gene in a patient with concomitant selective IgA deficiency who presented with a marked clinical improvement after vaccination against *S. pneumoniae*. This observation underlines the notion that vaccination against this microorganism is an important strategy for treatment of PID patients, particularly those presenting with increased susceptibility to infections caused by this agent.

**Electronic supplementary material:**

The online version of this article (doi:10.1007/s10875-012-9775-z) contains supplementary material, which is available to authorized users.

## Introduction

Primary immunodeficiencies (PIDs) are a heterogeneous group of inherited disorders of the immune system leading to enhanced susceptibility to infections [1]. The complement system is a crucial component of innate immunity and one of the main effector mechanisms of antibody-mediated immunity (reviewed in [2]).

Inherited complement deficiencies represent immunodeficiencies characterized by susceptibility to invasive infections by encapsulated bacteria such as *Streptococcus pneumoniae* (reviewed in [3–5]). The third component of the complement system (C3) is indispensable to all the known pathways of complement activation. C3 deficiency (OMIM*:* 120700) is a rare PID, leading to predisposition to recurrent pyogenic infections [1, 4].

A few biallelic defects in the *C3* gene have been described in patients suffering not only from *S. pneumoniae* infections [6–12] but also from autoimmune and immune-complex-related disorders, in particular affecting the kidney [13–15]. A similar phenotype can also be observed in patients with deficiency of complement factor H or I, respectively [5].

Here, we describe a patient with selective immunoglobulin A (IgA) deficiency presenting with recurrent airway infections caused by *S. pneumoniae* and bronchiectasis with no autoimmune or immune complex manifestations. Our molecular analyses revealed that the patient suffers from C3 deficiency caused by a novel, homozygous mutation in the *C3* gene.

## Methods

### Ethics Committee

This study has been approved by the Ethics Committee at the Medical University of Vienna, Austria. The patient and the other family members gave informed consent to the genetic analysis described here. Clinical data from the patients were provided in anonymized form by the responsible physician(s).

### Determination of Antibody Titers Against *Streptococcus pneumoniae*

Measurement of capsular polysaccharide serotype-specific immunoglobulin G (IgG) antibodies against *S. pneumoniae* was performed by enzyme-linked immunosorbent assay (ELISA) in serum samples before and 6 weeks after vaccination with *PNEUMO 23*® (unconjugated polysaccharide vaccine against *S. pneumoniae*) as described previously with minor modifications [16]. All sera were analyzed in duplicates in the same ELISA run. In order to eliminate antibodies to cell wall polysaccharides, microtiter plates were coated with capsular polysaccharide antigens (from the American Type Culture Collection (ATCC), Rockville, MD) and the samples were pre-incubated overnight with species-specific common cell wall polysaccharide from *S. pneumoniae* (CWPS; C-polysaccharide purified; Statens Serum Institute, Denmark). Antibody concentrations are indicated as the percentage of reference serum, the hyperimmune plasma pool (U.S. Pneumococcal Reference serum FDA7 CBER, Bethesda, MD) in units per milliliter (U/mL), where the reference plasma pool represents 100 U/mL for each serotype. Since patients with high pre-immunization titers may not generate a drastic increase after immunization, the final concentration of antibodies after immunization (regardless of increase from pre-immunization concentration) was taken into account. A minimal concentration of 20 U/mL in at least 50 % of the serotypes tested was considered as a positive response to the vaccination. This criterion was selected according to results obtained in 40 healthy Turkish children (age range: 5 to 15 years; median: 10 years and mean, 9.7 years) (O. Sanal, unpublished data).

### Molecular Analysis

Genomic DNA was isolated from whole blood obtained from the patient and parents using a commercially available kit (Wizard® Genomic DNA Purification Kit, Promega Corporation) according to the manufacturer’s instructions.

The primers used for sequencing of the *C3* gene were previously described by Goldberg et al. [10] with one additional pair covering part of exon 41 and the 3’UTR. This additional pair has the following sequences: forward 5′-ctcagctacatcatcgggaag-3′ and reverse 5′-ccttggctaaagaagtcagca-3′. All primers were purchased from Sigma Aldrich, Austria. Capillary sequencing was performed with the Big Dye Terminator v3.1 Cycle Sequencing Kit (Applied Biosystems, Germany) and analyzed on a 3130 × l Genetic Analyzer (Applied Biosystems). For sequence analysis, Sequencher DNA Software 4.10.1 (Gene Codes Corporation, USA) was used. The nucleotide variations found were further sequenced on both parents in order to evaluate the segregation. PolyPhen2 (Polymorphism Phenotyping v2, http://genetics.bwh.harvard.edu/pph2/) and SIFT (J. Craig Venter Institute, http://sift.jcvi.org/) were used to predict the effect of the mutation on protein function.

Phylogenetic conservation was assessed using protein sequences from Ensembl (http://www.ensembl.org) and UniProt (http://www.uniprot.org/) and aligned using UniProt multiple sequence alignment tool. For the protein modeling, we used Molsoft ICM Browser Pro software and a crystal structure model of the C3 convertase (2WIN) from the Protein Database website (http://www.rcsb.org/pdb/).

## Results

### Clinical Characterization of Patient and Family

**Fig. 1:**
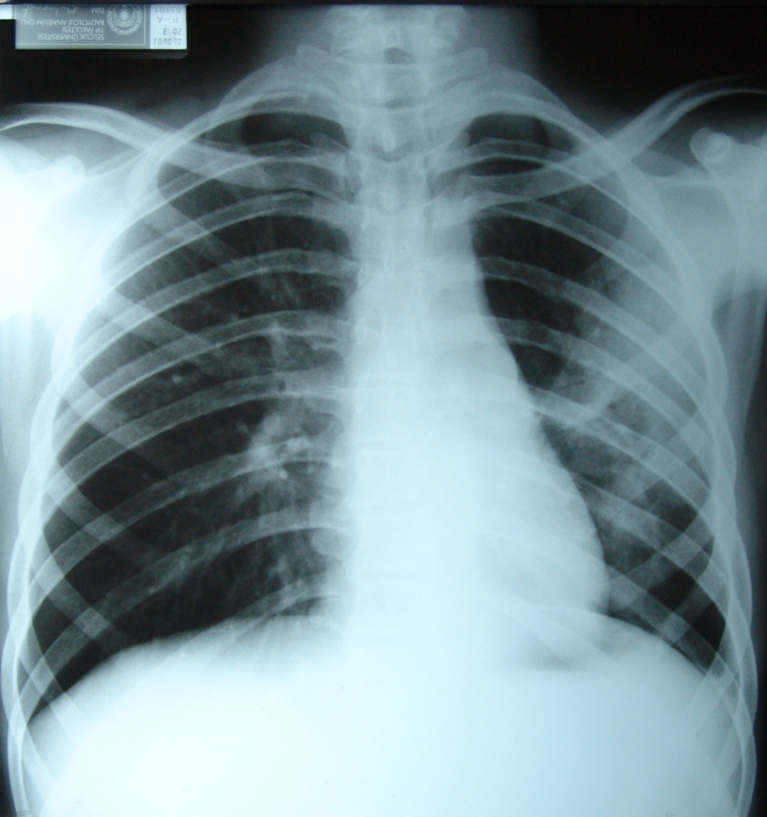
Lung radiography of the patient at admission showing pneumonia in the left lung and bronchial wall thickening on the right lung

At the age of 16 years, a male Turkish patient born to consanguineous parents (first-degree cousins) as the third of eight children, was admitted to hospital with a history of fever, cough and respiratory distress for 48 h. Physical examination revealed weight and height within normal range, a wound scar resulting from a thoracotomy performed at the age of 6 months, and crackles and bronchial respiratory sounds. A chest X-ray revealed pneumonia (Fig. 1) but no specific infectious agent could be isolated.

With regards to the past medical history, the patient had needed a tube thoracostomy due to pleural effusion at the age of 6 months and had suffered from recurrent lower respiratory infections with a frequency of 4 to 5 times per year. At the age of 8 years, bronchiectasis was detected and posterolateral segmentectomy in the left lower lobe was performed. Subsequently, the frequency of infections was reduced however, in a period of 6 years (from 14 to 20 years) he still presented with two episodes of otitis media and 8 episodes of lower respiratory tract infections for which hospitalization was necessary. At the age of 20 years, the patient was vaccinated for *S. pneumoniae* and has not suffered from severe infections or needed hospitalization since (7 years of follow up so far).

The patient has never presented with hematuria, hypertension or other clinical feature indicative of renal involvement or autoimmune disorder.

**Fig. 2:**
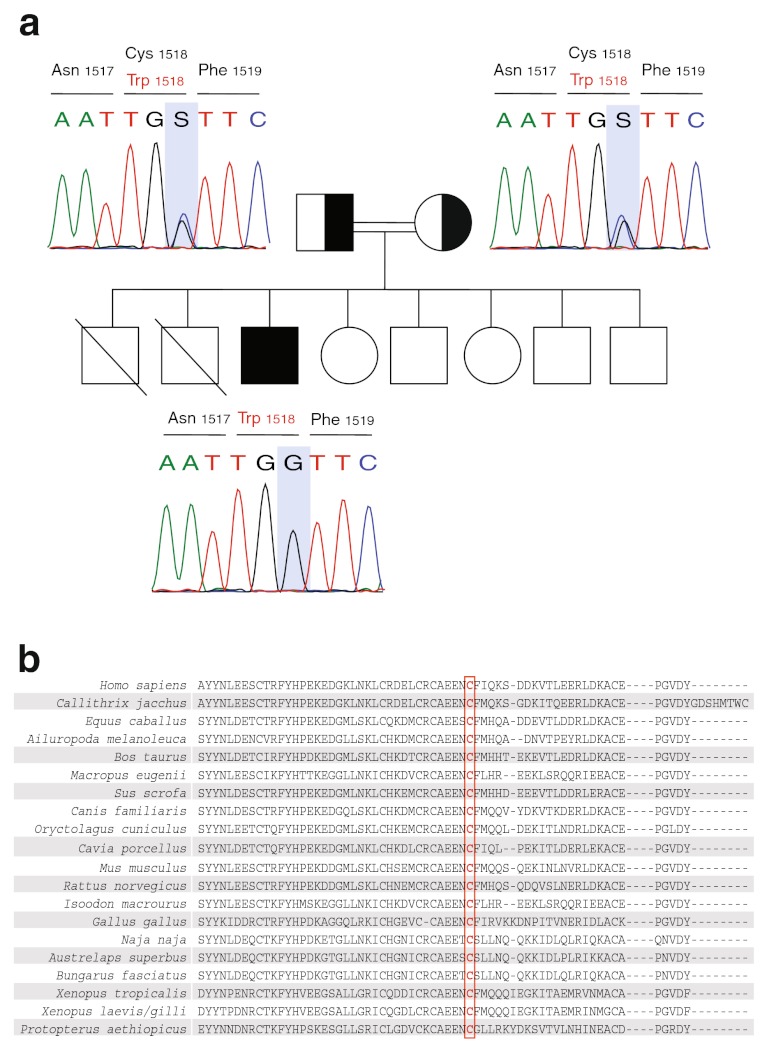
Pedigree and genetic analysis of the core family (*2a*) and phylogenetic conservation of the cysteine 1518 in C3 (*2b*). **a** Perfect segregation of the *C3* mutation (c. C4554G, p. Cys1518Trp) is shown in the patient and parents. Females are represented as *circles* and males as *squares*. *Filled and half-filled symbols* represent homozygous and heterozygous individuals, respectively. The point mutation is marked with a *grey bar*. The segregation in the siblings is not shown. **b** Phylogenetic conservation of the mutated amino acid in C3 in vertebrates. Cysteine 1518 is indicated with a *red box*

Notably, two of his brothers had died during the neonatal period for unknown reasons. His parents, three brothers and two sisters are healthy (see Fig. 2a and Supplementary Figure 1 for pedigrees).

### Laboratory Evaluation

The patient had normal red cell counts but elevated erythrocyte sedimentation rate (52 mm/h; reference values: 0–15 mm/h) and C-reactive protein levels (66 mg/dL; reference values: 8.5–10.6 mg/dL), respectively. Total leukocyte (7500/mm^3^) and lymphocyte counts (3500/mm^3^) were within the normal range. Routine urine analysis and renal function were unremarkable (see Table 1 for blood urea nitrogen and creatinine levels; urine analysis data are not shown). Immunoglobulin M, G and E levels were within the normal range as well as the IgG subclasses, while serum IgA levels were reduced (Table 1). As there were no signs or symptoms of autoimmunity, autoantibody levels were not evaluated.

**Table 1:** Description of the laboratory findings of the patient and parents

	Patient^1^	Father	Mother	Reference values
Blood urea nitrogen (mg/dL)	26			20–40
Creatinine (mg/dL)	0,8			0,6–1,2
CH50^a^ (U/mL)	0	71	71	>15
C3 levels (mg/dL)	8–19	63	71	90–180^2^
C4 levels (mg/dL)	20			10–40
IgA (mg/dL)	<5.8	230	341	44–244
IgG (mg/dL)	1811			640–2010
IgG1	1220			315–855
IgG2	668			64–495
IgG3	125			23–196
IgG4	25			11–157
IgM (mg/dL)	86			52–297
IgE (IU/mL)	46			0–100
Anti-A titer	1/64			1/10
Anti-B titer	1/128			1/10
Tuberculin test (mm)	10			5–10
In vitro PHA^b^ (%)	61			65.8 ± 9.2
Unstimulated NBT^c^ (%)	50			0–38
Stimulated NBT^c^ (%)	70			60–90
Total lymphocyte counts (cell/mm^3^)	3500			1700–5700
T lymphocytes (CD3+) (%)	68			55–79
(cells/mm^3^)	2040			1100–4100
T helper cells (CD4+) (%)	38			28–51
(cells/mm^3^)	1140			600–2400
Cytotoxic T cells (CD8+) (%)	26			16–42
(cells/mm^3^)	780			400–1500
Natural killer cells (CD16+56+) (%)	7			5–28
(cells/mm^3^)	210			200–1000
B lymphocytes (CD19+) (%)	20			10–28
(cells/mm^3^)	600			200–1400

^a^Total hemolytic complement activity test. ^b^ In vitro lymphocyte stimulation with 20 µg/mL of phytohemagglutinin for 72 h. The values refer to the percentages of the blastic transformation of lymphocytes (enlarged nucleus, condensed chromatin and/or with pores in the cytoplasm) evaluated using light microscopy. ^c^ Nitroblue tetrazolium semiquantitative test for evaluating neutrophil oxidative burst, values refer to the percentages of activated neutrophils presenting with respiratory burst activity

^1^Patient examination at the age of 16 years

^2^Normal values in age-matched Turkish subjects

Six weeks after vaccination for *S. pneumoniae* (20 years of age), the patient demonstrated a positive antibody response (Table 2).

**Table 2:** Specific IgG antibody levels in the patient before and after *S. pneumoniae* vaccine (U/mL). Reference values: ≥20 U/mL

Serotypes	3	6B	14	19F	23F	7F
Before vaccination	22	16	32	19	16	18
6 weeks after vaccination	29	>100	46	65	68	20

C3 levels were severely decreased in the patient, varying from 8 to 19 mg/dL at 6 different measurements (Table 1; reference values: 90–180 mg/dL), with absent complement hemolytic activity measured using the CH50 test. The other complement levels (C1, C2, C4, C5, C6, C7, C8, C9, Factor H and Factor I) were found to be normal (see Table 1 for C4; other data not shown). The proliferative response of peripheral blood lymphocytes to phytohemagglutinin and the spontaneous and stimulated nitroblue tetrazolium test were normal (Table 1). The parents showed normal levels of IgA and normal CH50, but reduced C3 levels (63 mg/dL and 71 mg/dL), respectively. Details of the laboratory findings of the patient and his parents are shown in Table 1.

### Mutation Identification in the *C3* Gene

**Fig. 3:**
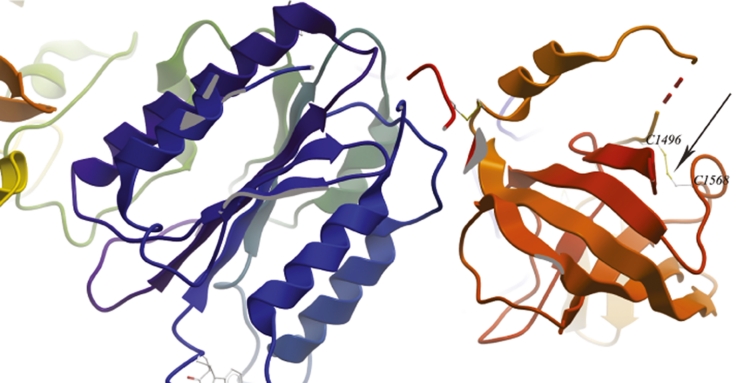
Details of the structure of C345C domain of C3 and factor B based on the PDB model 2WIN. The figure shows the C345C domain on the *right hand side* (in red/orange) and factor B on the *left hand side* (in blue/green). Note that, in the PDB model, the residues Cys1518 and Cys1590 correspond to Cys1496 and Cys 1568, respectively. The arrow points the disulphide bridge formed by both cysteine residues

The patient was evaluated for an underlying mutation in the *C3* gene. Sanger sequencing identified a homozygous missense mutation in exon 38 (c. C4554G, p. Cys1518Trp). Molecular segregation analysis showed perfect segregation, with both parents and three siblings being carriers of one affected allele (Fig. 2a and Supplementary figure 1). To date, this variant has neither been annotated as a polymorphism in NCBI (http://www.ncbi.nlm.nih.gov/), nor in UCSC Genomic Bioinformatics site (http://genome.ucsc.edu/) or Ensembl (http://www.ensembl.org), respectively. Polyphen2 and Sorting Intolerant from Tolerant (SIFT) analysis predict that the substitution of cysteine to tryptophan at position 1518 of C3 is very likely to affect protein function, with the maximum score in both analyses (1.000 in Polyphen2 and 0.000 in SIFT, respectively). As indicated in Fig. 2b, the cysteine residue at position 1518 is conserved among vertebrates [17] and it has been shown to form a disulfide bridge with cysteine 1590 [17, 18]. In Fig. 3, a detail of the C345C domain in complex with Factor B (model based on the PDB 2WIN) is depicted and the localization of the disulphide bridge is shown.

## Discussion

The complement system is a protein network crucial for both innate and adaptive immune responses. C3 is the convergence point for all the known complement activation cascades, resulting in cleavage of C3 into C3a (anaphylatoxin) and C3b (reviewed in [2]). The latter is an important product for opsonization of bacteria including encapsulated bacteria such as *S. pneumoniae* [18, 19], for amplification of complement activation through the alternative pathway, where the association of C3b with complement factor B is essential, and for cell lysis through the formation of C5 convertase (reviewed in [2]). Patients with C3 deficiency frequently develop severe episodes of recurrent pneumonia, meningitis or sepsis. Clinically, these patients present at an early age with overwhelming infections caused by *S. pneumoniae* [4, 5]. Autoimmunity and other immune manifestations, frequently affecting the kidney, are also observed in C3-deficient patients [13–15].

Since the parents of the patient described here were first-degree cousins and the index patient’s severe clinical manifestations were unlikely to be explained by the diagnosis of isolated IgA deficiency, an autosomal recessive disorder was suspected. We discovered and here describe a novel, homozygous missense mutation in *C3,* altering a highly conserved amino acid found in the first position of the C345C domain of the C3 protein which is hypothesized to function as a binding site for factor B, as required for C3 convertase formation [20–22]. This domain is known to undergo large rearrangements upon activation and is present in the C3b molecule [23, 24]. Furthermore, this cysteine residue forms one of the disulfide bonds in the C3 protein [20, 25], thus its loss will likely affect protein folding and/or stability.

After vaccination for *S. pneumoniae*, our patient showed a marked clinical improvement. In line with this observation, it has been shown that mice depleted for C3 by intraperitoneal injection of cobra venom factor which are immunized against *S. pneumoniae* have reduced sepsis when colonized with this bacterium compared to control or neutrophil-depleted mice [26]. Previous studies have illustrated that C3-deficient patients are able to mount adaptive immune responses to conjugated vaccines against *S. pneumoniae* [8, 9].

As mentioned, in addition to C3 deficiency the patient showed selective IgA deficiency (sIgAD). sIgAD is the most common form of primary immunodeficiency defined by decreased levels of IgA in the presence of normal levels of other immunoglobulin isotypes (reviewed in [27]). Patients are predisposed to recurrent sinopulmonary infections, gastrointestinal disorders, autoimmune diseases, atopy and malignancies [27, 28]. Amongst the gastrointestinal disorders, giardiasis, malabsorption, lactose intolerance, celiac disease, ulcerative colitis and nodular lymphoid hyperplasia can be found [27]. Respiratory tract infections are the most frequent morbidities in sIgAD patients [28], however, bronchiectasis is a rare complication [29]. Although sIgAD is often asymptomatic, patients with concomitant IgG2 deficiency may present with impaired antibody responses against polysaccharide antigens and show predisposition to more severe bacterial infections [28].

Besides C3 deficiency, other deficiencies of adaptive or innate immunity can also lead to increased susceptibility to infections caused by *S. pneumoniae*, albeit with differences in the clinical and laboratory findings (reviewed in [4, 12]). Two interesting examples of such innate immune deficiencies are IRAK4 and MYD88 deficiency, respectively. IRAK4- or MYD88-deficient patients are predisposed to recurrent invasive infections with *S. pneumoniae*, especially meningitis (reviewed in [12]). These patients also frequently present with impaired ability to increase plasma C-reactive protein and to mount fever in response to infection, with spontaneous improvement in adolescence (reviewed in [12]). By contrast, our patient presented mainly with pneumonias, and similar to other C3-deficient patients [4, 5], he showed high levels of CRP and had episodes of fever, with infectious episodes persisting throughout adolescence.

Taken together, the following observations support our hypothesis that the clinical phenotype of our patient was - at least predominantly - caused by the underlying deficiency in C3 rather than associated with sIgAD: 1) sinusitis or gastrointestinal disorders are absent in the patient; 2) bronchiectasis is observed although our patient presented with normal IgG2 levels and normal antibody responses to polysaccharide antigens; 3) the patient displays a marked and relatively specific susceptibility to infections with encapsulated bacteria such as *S. pneumoniae*; and 4) there was a marked clinical amelioration upon vaccination against *S. pneumoniae*.

## Conclusions

We here report a novel homozygous mutation in *C3* in a patient with recurrent and severe infections caused by *Streptococcus pneumoniae* and associated IgA deficiency. The case presented here highlights the importance of a more thorough evaluation of sIgAD patients when the clinical presentation is unusual or more severe than expected. In case of severe infections caused by encapsulated agents such as *S. pneumoniae*, a careful evaluation of complement components is mandatory. Our data lend further support to the concept that vaccination against this microorganism is critical for immunodeficient patients, in particular for inherited complement deficiencies.

## Electronic supplementary material

Below is the link to the electronic supplementary material.

Supplementary Fig. 1 Extended family pedigree including five generations. The *C3* mutation (c. C4554G, p. Cys1518Trp) shows perfect segregation with the disease. Females are depicted in circles and males in squares. The filled symbol represents the homozygous affected individual (IV-6) and half-filled symbols represent heterozygous individuals. Homozygous healthy subjects are represented as empty symbols. Crossed-out symbols denote deceased individuals. Subjects that were not sequenced are illustrated in grey. Note that three siblings, one nephew and one niece of the affected subject are also heterozygous for the *C3* mutation. (GIF 289 kb)

High resolution (EPS 289 kb)

## Abbreviations

C3: Complement component 3
DNA: Deoxyribonucleic acid
ELISA: Enzyme-linked immunosorbent assay
IgA: Immunoglobulin A
IgG: Immunoglobulin G
PID: Primary immunodeficiency
